# Long- and very long-chain ceramides are predictors of acute kidney injury in patients with acute coronary syndrome: the PEACP study

**DOI:** 10.1186/s12933-023-01831-6

**Published:** 2023-04-20

**Authors:** Lianjing Liang, Dongze Li, Rui Zeng, Haihong Zhang, Lin Lv, Wei Wei, Zhi Wan

**Affiliations:** Present Address: Department of Emergency Medicine, Disaster Medical Center, West China School of Medicine, West China Hospital, Sichuan University, Chengdu, China

**Keywords:** Ceramides, Acute kidney injury, Acute coronary syndrome, Percutaneous coronary intervention

## Abstract

**Background:**

Acute kidney injury (AKI) can be caused by multiple factors/events, including acute coronary syndrome (ACS). Ceramides are involved in atherosclerosis progression, cardiovascular events, and renal damage. Almost no studies have been conducted on the relationship between ceramide concentrations and AKI events. Therefore, we evaluated the association between plasma ceramide level at admission and AKI in patients with ACS undergoing percutaneous coronary intervention.

**Methods:**

We enrolled 842 ACS patients from the Prospective Multicenter Study for Early Evaluation of Acute Chest Pain. AKI was defined using the criteria from the 2012 Kidney Disease: Improving Global Outcomes. Eleven C16–C26 ceramides were measured using the high-performance liquid chromatography interfaced to tandem mass spectrometer procedure. Logistic regression models were used to evaluate relationships between ceramides and AKI risk. The area under the receiver operating characteristic curves (AUC) was used to evaluate differences between ceramides.

**Results:**

Overall, 139 (16.5%) patients developed AKI during hospitalisation. Patients who developed AKI had higher levels of Cer(d18:1/16:0), Cer(d18:1/18:0), Cer(d18:1/20:0), Cer(d18:1/21:0), Cer(d18:1/24:1), and Cer(d18:1/24:2) than patients who did not (P < 0.05). In risk-factor adjusted logistic regression models, these ceramides were independently associated with AKI risk (P < 0.05). Cer(d18:1/24:2) had the highest odds ratio of 3.503 (Q4 vs. Q1, 95% confidence interval: 1.743–7.040, P < 0.001). Ceramides had AUCs of 0.581–0.661 (P < 0.001) for AKI. Each ceramide combined with the Mehran risk score (AUC: 0.780) had AUCs of 0.802–0.808, greater than the Mehran risk score alone.

**Conclusion:**

Long-chain and very-long-chain ceramide levels may help determine the high AKI risk beyond traditional assessments.

**Supplementary Information:**

The online version contains supplementary material available at 10.1186/s12933-023-01831-6.

## Introduction

Acute kidney injury (AKI) is a complex syndrome that is wildly considered a common and catastrophic complication of acute coronary syndrome (ACS) [1]. The presence of AKI is related to worsening prognosis at both short- and long-term follow-up, raising mortality two- to three-fold [2–5]. The incidence of ACS-related AKI is heterogeneous at 5–55% and varies with the AKI diagnostic criteria, clinical background, and patient population [1]. Given the high incidence of AKI in ACS and the lack of specific therapy, discovering novel biomarkers is of great significance for more effective clinical prediction and prevention.

Ceramide is a type of complex sphingolipid; it is at the centre of sphingolipid synthesis and degradation metabolism. Ceramide consists of two hydrocarbon chains: the long-chain base of sphingosine and the saturated, or unsaturated, fatty acid of the C16–C26 chain length, linked to the C-2 position of the long-chain base of sphingosine via N-acylation [6]. In the blood, ceramides constitute a part of the circulating lipoprotein particles and are also present in blood cells [6]. Multiple studies have repeatedly confirmed the strong predictive value of plasma ceramides for cardiovascular events and mortality in patients with stable and unstable coronary heart disease [7–11], owing to their biochemical role in atherosclerosis progression and rupture of plaque [12]. A potential detrimental impact of a higher ceramide ratio (C16:0/24:0) on cardiac remodelling has been reported [13]. Importantly, ceramides have strong biological activity, play a central role in inflammatory signalling, cellular stress response, cell membrane integrity, nephrotoxicity, and apoptosis, and are associated with worsening cardiac function [14–16]; these mechanisms are a key component of AKI pathogenesis [1, 17]. Ceramide synthases 5 and 6 are upregulated by stress stimuli and promote the synthesis of long-chain ceramides, which are involved in various metabolic dysfunctions [18–24]. On the other hand, very long-chain ceramides are considered benign or protective [25, 26]. Therefore, an increased ratio of Cer(d18:1/24:2) indicates an increase in long-chain ceramides and/or a decrease in benign/protective ceramides, suggesting a shift toward metabolic dysfunction. Long-chain ceramides are known to be involved in diabetic kidney disease [27]. It is why this study selected long-chain and very-long-chain ceramides.

Recent studies have shown that increased levels of plasma ceramides are associated with chronic kidney disease, independent of common cardiovascular risk factors [28, 29]. One study suggested that the increased accumulation of ceramides in plasma plays an important role in kidney injury [30]. To our knowledge, almost no studies have been conducted on the relationship between ceramide concentrations and AKI events. We hypothesised that an increased plasma ceramide concentration results in a higher risk of AKI, especially in vulnerable populations, such as patients with ACS. Therefore, this Prospective Multicenter Study for Early Evaluation of Acute Chest Pain (PEACP) aimed to investigate the relationship between plasma ceramide levels and the risk of AKI in patients with ACS undergoing percutaneous coronary intervention (PCI).

## Methods

### Study design

The PEACP study was conducted to investigate new markers of diagnosis and prognosis for patients with acute chest pain using multiomics data by mass spectrometry detection. The PEACP study enrolled patients with acute chest pain from the chest pain centres of seven tertiary hospitals in China; it is registered at https://clinicaltrials.gov/ct2/home (Identifier: NCT04122573). This study was designed to evaluate whether the level of ceramides could predict AKI in patients with ACS undergoing primary PCI.

### Study population

From November 2019 to April 2020, 3 610 patients with chest pain onset time < 24 h visited the acute chest pain centre: 923 patients were diagnosed with ACS and underwent PCI according to the diagnostic standard for non-ST-elevation ACS (NSTE-ACS) and ST-elevation myocardial infarction (STEMI), according to the American College of Cardiology and American Heart Association guidelines [31, 32]. The inclusion criteria included: diagnosed as ACS; > 18 years old; onset time < 24 h; signed the informed consent. The exclusion criteria were received thrombolysis, unqualified data of ceramides, lack of dynamic assessment of creatinine, and requiring chronic haemodialysis treatment. Finally, 842 patients were included in this study. (Additional file 1: Figure 1).

### Data collection and definition

Data were recorded prospectively by two independent researchers, including vital signs, chronic disease history, inpatient medications, imaging examination, and lab tests. Leukocytes, platelets, and hemoglobin was measured on a haematology analysis system (LH 750, Beckman Coulter, Brea, CA). Serum creatinine, estimated glomerular filtration rate (eGFR), blood lipid, and blood glucose were conducted using the Architect c16000 analyser (Abbott Diagnostics, Dallas, TX). Cardiac biomarkers were measured using an immunology analyser (Cobas E601, Roche Diagnostics). The Global Registry of Acute Coronary Events (GRACE) [33] score and Gensini score [34] were recorded as former reports [35, 36]. The Mehran risk score includes the following components: use of intra-aortic balloon pump, age, anaemia, diabetes mellitus, congestive heart failure, contrast media volume, hypotension, and eGFR [37]. If there was a difference between the two data checks, another researcher participated in the judgment.

Diagnosis and staging of AKI were established using the Kidney Disease: Improving Global Outcomes (KDIGO) standard [38]. Meeting any of the following conditions is considered AKI stage 1: elevated serum creatinine level > 0.3 mg/dL (26.5 mmol/L) less than 2 days; serum creatinine increase to 1.5–1.9-fold from the baseline level; urine output < 0.5 mL/kg/h for 6–12 h. Meeting any of the following conditions is considered AKI stage 2: serum creatinine increase to 2.0–2.9-fold from the baseline level; urine output < 0.5 mL/kg/h for 12 h; Meeting any of the following conditions is considered AKI stage 3: serum creatinine concentration > 4.0 mg/dL (353.6 mmol/L); serum creatinine increased to > 3.0-fold from the baseline level; urine output < 0.3 mL/kg/h for 24 h; anuria for 12 h. The baseline level was regarded as the admission serum creatinine value tested at the emergency department. The primary endpoint of this study was AKI development.

### Blood sample collection and quantification of ceramides

Blood samples (5 ml) were collected in EDTA tubes and stored at 4 °C for 30 min. To obtain plasma, blood samples were centrifuged at 1500 rpm for 10 min at 4 °C and then stored at −80 °C until further testing. Quality control (QC) was ensured by taking a mixture of 2 μL from each study sample. Ten microlitres of plasma were spiked before extraction with deuterated internal standards. The extraction of ceramide was performed as described previously [39]. Briefly, samples (150 µL) were deproteinated with 450 µL of cold isopropanol, vortexed for 30 s, centrifuged at 12,000 × *g* at 4 °C for 5 min, and 180 µL of the supernatant was transferred to an EP tube. The high-performance liquid chromatography interfaced with a tandem mass spectrometer (LC–MS/MS) procedure for ceramides testing has been showed in Supplementary materials in detail as reported previously [40]. The individual ceramides were quantified on the TSQ Quantiva Triple Quadrupole mass spectrometer equipped with the Dionex Ultimate 3000 UHPLC system (Thermo Fisher, San Jose, CA) and working in the MRM mode. Quantification was assessed using calibration line samples prepared with known amounts of synthetic Cer(d18:1/16:0), Cer(d18:1/18:0), Cer(d18:1/20:0), Cer(d18:1/21:0), Cer(d18:1/22:0), Cer(d18:1/23:0), Cer(d18:1/24:0), Cer(d18:1/24:1), Cer(d18:1/24:2), Cer(d18:1/25:0), and Cer(d18:1/26:0) and corresponding deuterated (D7) standards. The peak area ratios of ceramides and their deuterated forms were read. The value of ceramide added was plotted, and then linear regression analysis was carried out.

The endogenous plasma ceramide concentrations were derived from the obtained individual regression equations by calculating corresponding concentrations from the measured peak area ratios in samples. The precision (coefficient variance) and accuracy (relative error) for intra and inter for all 11 ceramides were within 10%; these were determined as previously described [40]. The final ceramide concentrations in plasma are presented as µmol/L.

### Statistical analysis

Based on our pilot study, the incidence of AKI is approximately 15%. It is assumed that the area under the receiver operating characteristic curve (AUC) of ceramides for AKI is > 0.6, and to satisfy this difference with 80% power at 5% significance (2-tailed), a sample size of 546 is required. The sample size was calculated using MedCalc Statistical Software version 19.0.2 (MedCalc Software, Belgium).

Categorical variables are reported as frequency (%) and were compared by the χ^2^ test. Nonparametric continuous variables are expressed as medians (25th–75th) and compared using the Mann–Whitney U test. Parametric continuous variables are performed as means ± standard deviations and were compared using analysis of variance.

The correlation between all 11 ceramides and common prognostic factors of ACS was determined using Pearson’s correlation analysis. Logistic regression models were used to evaluate whether the levels of ceramides were associated with the risk of AKI after adjustment for confounding factors, including age, sex, systolic blood pressure, heart rate, body mass index, statin use, hypertension, diabetes, hyperlipidemia, Killip class, white blood cell count, low-density lipoprotein cholesterol, total cholesterol, triglyceride, estimated glomerular filtration rate, high sensitive cardiac troponin T, N-terminal pro-brain natriuretic peptide, and Mehran risk score. The odds ratio was calculated per the standard deviation (SD) and interquartile range of ceramides. AUC was determined to assess the predictive power of the Mehran risk score, all ceramides, and the ratio of all ceramides to Cer(d18:1–24:0) for AKI and to investigate the incremental predictive value of ceramides over the Mehran risk score. A two-tailed *p* < 0.05 indicated statistical significance. Data were analysed by SPSS Statistics (version 26.0, Chicago, IL) and R (version 3.5.1, Vienna, Austria).

## Results

### Baseline characteristics

**Fig. 1:**
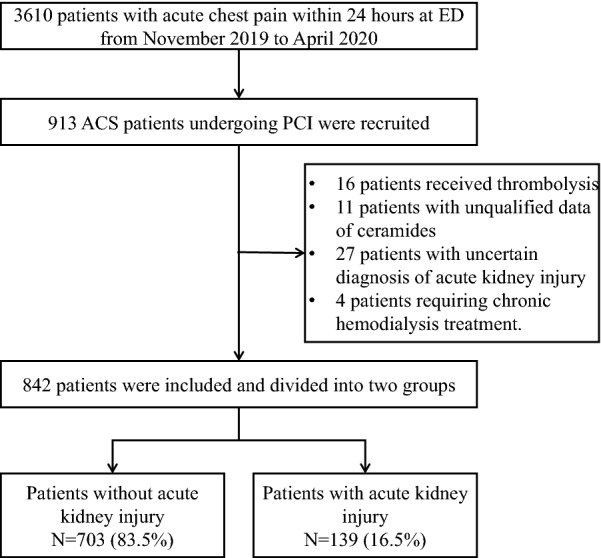
Study flow-process diagram. *ED* emergency department, *PCI* percutaneous coronary intervention

Among the 842 ACS patients undergoing PCI, 620 (73.6%) were men, and the average age was 66.9 ± 13.0 years. During the hospitalisation period, there were 75 (8.9%), 31 (3.7%), and 33 (3.9%) patients who developed AKI stages 1, 2, and 3, respectively. Table 1 shows the baseline characteristics of patients classified as having non-AKI, AKI stages 1 to 3. Generally, patients with AKI were older and more likely to have hypertension and diabetes; higher Killip class, heart rate, white blood cell count, neutrophil cell count, fibrinogen, blood glucose, creatinine, eGFR; NT-proBNP, hs-CTNT, contrast dose, GRACE score, Gensini score, Mehran risk score; and lower haemoglobin (*p* < 0.05) than patients without AKI (Fig. 1).

**Table 1:** Baseline clinical characteristics of patients grouped by acute kidney injury

Variables	Non-AKI	AKI stage 1	AKI stage 2	AKI stage 3	P
N	703 (83.5)	75 (8.9)	31 (3.7)	33 (3.9)	
Age, years	65.0 ± 12.6	74.8 ± 11.2	76.2 ± 9.3	69.2 ± 15.7	< 0.001
Males, n (%)	177 (25.2)	22 (29.3)	12 (38.7)	11 (33.3)	0.250
BMI, kg/m^2^	24.2 ± 2.7	23.4 ± 2.3	23.7 ± 1.6	23.8 ± 2.6	0.066
Smoking, n (%)	328 (46.7)	33 (44)	9 (29)	12 (36.4)	0.176
Drinking, n (%)	181 (25.9)	15 (20)	7 (22.6)	10 (30.3)	0.619
Hypertension, n (%)	391 (55.6)	47 (62.7)	22 (71)	27 (81.8)	0.007
Diabetes, n (%)	193 (27.5)	33 (44)	16 (51.6)	16 (48.5)	< 0.001
Hyperlipidemia, n (%)	72 (10.2)	8 (10.7)	2 (6.5)	4 (12.1)	0.890
SBP, mmHg	132.4 ± 23.4	130.4 ± 29.1	132.6 ± 29.9	142.3 ± 29.3	0.116
DBP, mmHg	81 ± 15.1	78.2 ± 20	74.8 ± 21.3	83.9 ± 24	0.061
Heart rate, /min	78.8 ± 16.1	85.1 ± 25	82.5 ± 25.7	85.7 ± 17.5	0.003
Killip class ≥ 1, n (%)	315 (44.8)	43 (57.3)	23 (74.2)	17 (51.5)	0.003
STEMI, n (%)	303 (43.1)	32 (42.7)	13 (41.9)	10 (30.3)	0.548
Laboratory findings					
WBC, 10^9/L^	9.0 ± 3.3	10.5 ± 5.5	11.8 ± 4.1	8.9 ± 3.9	< 0.001
Neutrophil, 10^9^/L	6.8 ± 3.2	8.3 ± 5.2	9.5 ± 4	7.1 ± 3.6	< 0.001
Hemoglobin, g/L	136.3 ± 19.5	120.8 ± 22.9	113.8 ± 23.2	101.8 ± 25.5	< 0.001
Platelet count, 10^9/L^	179 (142–226)	182 (133–217)	219 (126–273)	181 (145–246)	0.638
Fibrinogen, g/L	3.5 ± 1.3	3.9 ± 1.4	4.1 ± 1.2	5.2 ± 1.9	< 0.001
Blood glucose, mmol/L	8.8 ± 4.2	11.1 ± 5.8	11.4 ± 7.3	9.8 ± 4.4	< 0.001
Creatinine, μmol/L	76 (64–88)	129 (119–150)	195 (167–210)	327 (250–586)	< 0.001
eGFR	85.6 ± 18.1	42.6 ± 6.7	29.6 ± 15.3	19.4 ± 22.5	< 0.001
BUN, mmol/L	5.3 (4.4–6.7)	8.9 (7.3–12.2)	13.9 (10.9–16.3)	19 (13.5–25.4)	< 0.001
Triglycerides, mmol/L	1.49 (1.05–2.18)	1.33 (0.98–2.02)	1.41 (1.19–2.04)	1.68 (1.24–2.53)	0.290
Total cholesterol, mmol/L	4.4 ± 1.2	4.2 ± 1	4.3 ± 1.5	4.4 ± 1.7	0.710
HDL-C, mmol/L	1.1 ± 0.3	1.1 ± 0.4	1.2 ± 0.4	1.1 ± 0.4	0.541
LDL-C, mmol/L	2.7 ± 1	2.5 ± 0.8	2.6 ± 1.3	2.6 ± 1.4	0.327
NT-proBNP, pg/mL	685 (180–2317)	4585 (1344–12382)	7530 (3711–22358)	13281 (4382–35000)	< 0.001
Hs-CTnT,pg/mL	308 (38–1385)	568 (54–2555)	1287 (616–4284)	914 (185–4961)	< 0.001
Admission medication					
Aspirin, n (%)	691 (98.3)	73 (97.3)	29 (93.5)	31 (93.9)	0.116
Dual antiplatelet, n (%)	638 (90.8)	68 (90.7)	29 (93.5)	26 (78.8)	0.134
β receptor blockers, n (%)	404 (57.5)	27 (36.0)	15 (48.4)	17 (51.5)	0.004
Statin, n (%)	660 (93.9)	56 (74.7)	25 (80.6)	29 (87.9)	< 0.001
ACEI or ARB, n (%)	285 (40.5)	18 (24.0)	9 (29.0)	7 (21.2)	0.004
Percutaneous coronary intervention					
Contrast dose, ml	98.8 ± 14.5	112.9 ± 12.5	132.9 ± 15.7	147.3 ± 21.6	< 0.001
Stent ≥ 2, n (%)	207 (32.9)	19 (31.7)	13 (50)	13 (41.9)	0.227
Three-vessel disease, n (%)	352 (50.1)	35 (46.7)	14 (45.2)	21 (63.6)	0.380
Risk score					
GRACE score	135 ± 34.1	151.9 ± 36.3	173.1 ± 39.5	182.8 ± 40.0	< 0.001
Gensini score	51 (24–79)	58 (17–82)	68 (43–101)	89 (45–68)	0.020
Mehran risk score	9.6 ± 4.8	14 ± 4.8	15.2 ± 4.7	16.2 ± 4.4	< 0.001

*AKI* acute kidney injury, *SBP* systolic blood pressure, *DBP* diastolic blood pressure, *BMI* body mass index, *STEMI* ST-elevation elevated myocardial infarction, *WBC* white blood cell count, *BUN* Blood urea nitrogen, *HDL-C* high-density lipoprotein cholesterol, *LDL-C* low-density lipoprotein cholesterol, *eGFR* estimated glomerular filtration rate, *hs-CTnT* high sensitive cardiac troponin T, *NT-proBNP* N-terminal pro-brain natriuretic peptide, *GRACE* Global Registry of Acute Coronary Events

### Correlation of all ceramides and common risk factors for ACS

**Fig. 2:**
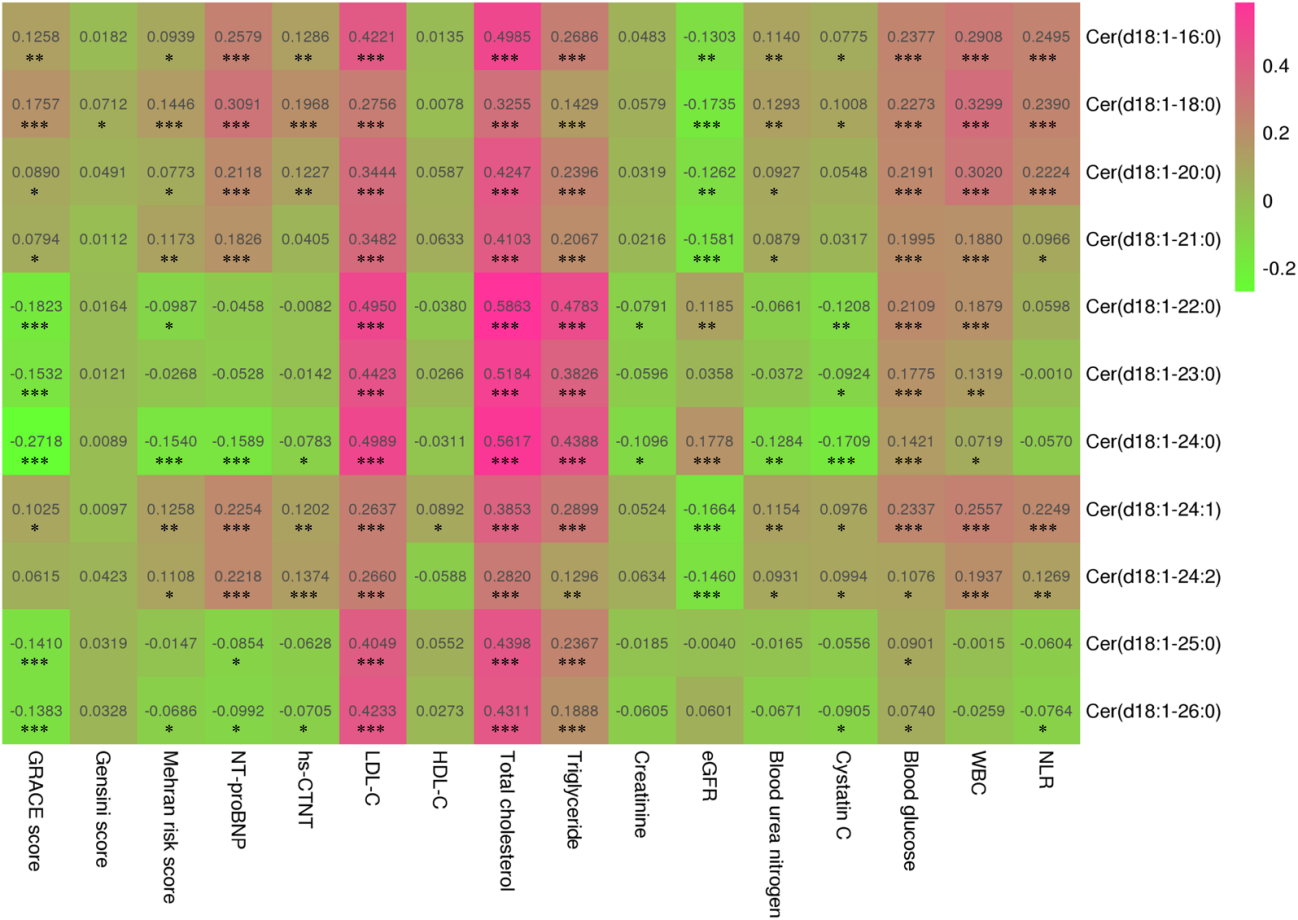
Correlation between all eleven ceramides and common prognostic factors in patients with acute coronary syndrome. The value in the heat map is the correlation coefficient. * < 0.05; ** < 0.001; *** < 0.0001. *GRACE* Global Registry of Acute Coronary Events, *WBC* white blood cell count, *BUN* Blood urea nitrogen, *HDL-C* high-density lipoprotein, *LDL-C* low-density lipoprotein, *eGFR* estimated glomerular filtration rate, *hs-CTnT* high sensitive cardiac troponin T, *NT-proBNP* N-terminal pro-brain natriuretic peptide, *NLR* neutrophil to lymphocyte ratio

According to Spearman’s correlation analysis (Fig. 2), all ceramides had a significant positive correlation with blood lipids (cholesterol and triglycerides) (P < 0.0001), while Cer(d18:1/16:0), Cer(d18:1/18:0), Cer(d18:1/20:0), Cer(d18:1/21:0), Cer(d18:1/24:1), and Cer(d18:1/24:2) had a significant positive correlation with ACS risk score (GRACE score and Mehran risk score), cardiac markers (NT-proBNP and hs-CTNT), baseline renal function (creatinine, eGFR, and blood urea nitrogen), stress marker (blood glucose), and inflammatory markers (white blood cell count and neutrophil to lymphocyte ratio). Moreover, all 11 ceramides had a positive correlation with each other (P < 0.0001, Additional file 1: Figure S1A). We calculated the average mass fraction of these ceramides in ACS patients; Cer(d18:1/24:0) and Cer(d18:1/24:1) accounted for the smallest (5%) and largest (20%) mass fractions, respectively (Additional file 1: Figure S1B).

### Relationship between all ceramides and AKI

Medians and interquartile ranges of levels of ceramides in patients with and without AKI are shown in Table 2. Patients with AKI had higher levels of Cer(d18:1/16:0), Cer(d18:1/18:0), Cer(d18:1/20:0), Cer(d18:1/21:0), Cer(d18:1/24:1), and Cer(d18:1/24:2) and a lower level of Cer(d18:1/24:0) than patients without AKI (P < 0.05).

**Table 2:** Medians and interquartile ranges of ceramides in patients with and without acute kidney injury (AKI)

Ceramides	Non-AKI	AKI	*P*
Cer(d18:1–16:0)	1.394 (1.092–1.766)	1.656 (1.200–2.231)	< 0.001
Cer(d18:1–18:0)	1.989 (1.333–2.722)	2.411 (1.768–3.931)	< 0.001
Cer(d18:1–20:0)	1.952 (1.512–2.564)	2.160 (1.585–3.447)	0.003
Cer(d18:1–21:0)	1.480 (1.061–1.966)	1.778 (1.264–2.458)	< 0.001
Cer(d18:1–22:0)	1.094 (0.804–1.446)	1.036 (0.742–1.448)	0.459
Cer(d18:1–23:0)	0.849 (0.618–1.178)	0.870 (0.634–1.192)	0.452
Cer(d18:1–24:0)	0.837 (0.587–1.137)	0.725 (0.528–1.031)	0.011
Cer(d18:1–24:1)	2.932 (2.025–3.963)	3.645 (2.426–5.496)	< 0.001
Cer(d18:1–24:2)	0.785 (0.541–1.164)	1.051 (0.765–1.614)	< 0.001
Cer(d18:1–25:0)	0.749 (0.497–1.052)	0.736 (0.479–1.109)	0.996
Cer(d18:1–26:0)	0.832 (0.526–1.239)	0.803 (0.435–1.187)	0.458

*AKI* acute kidney injury

**Fig. 3:**
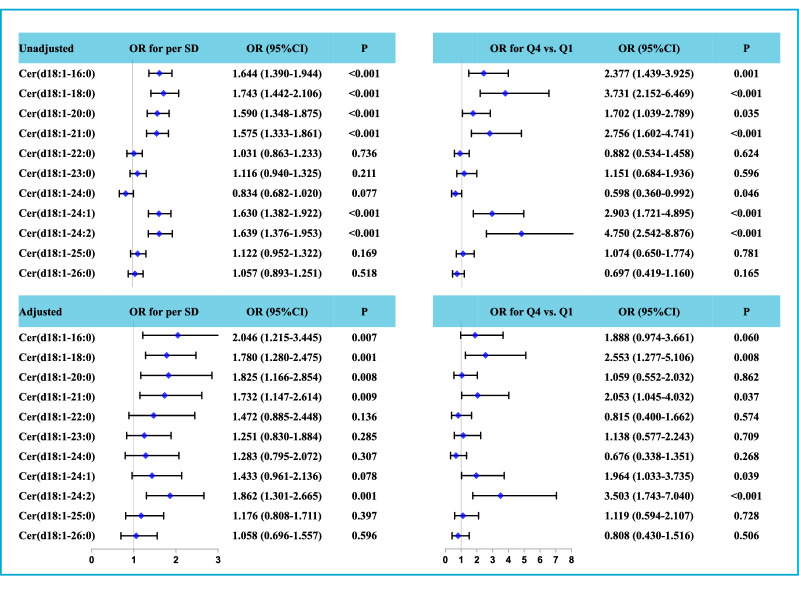
Unadjusted and adjusted OR of eleven ceramides for AKI. Models were adjusted by age, sex (male vs. female), systolic blood pressure, heart rate, body mass index, statin (yes vs. no), hypertension (yes vs. no), diabetes (yes vs. no), hyperlipidemia (yes vs. no), Killip class (I vs. II vs. III vs. IV), white blood cell count, low-density lipoprotein cholesterol, total cholesterol, triglyceride, estimated glomerular filtration rate, high sensitive cardiac troponin T, N-terminal pro-brain natriuretic peptide, and Mehran risk score. *OR* odds, ratio, *CI* confidence interval, *AKI* acute kidney injury

Figure 3 shows non-adjusted and adjusted ORs for all 11 ceramides. Cer(d18:1/16:0), Cer(d18:1/18:0), Cer(d18:1/20:0), Cer(d18:1/21:0), Cer(d18:1/24:1), and Cer(d18:1/24:2) were independently associated with the risk of AKI (P < 0.05). Among them, Cer(d18:1/16:0) had the highest adjusted OR (2.046, 95% confidence interval [CI] 1.215–3.445, P = 0.007) for per SD increase, and Cer(d18:1/24:2) had the highest adjusted OR (3.503, 95% CI 1.743–7.040, P < 0.001) when Q4 was compared with Q1. The multivariable logistic regression analysis results were consistent for patients with NSTE-ACS and STEMI (Additional file 2: Table S1). We also analyzed the ratios of all 10 ceramides to Cer(d18:1/24:0). As shown in Additional file 2: Table S2, all 10 ratios were independently associated with AKI.

### Predictive value of ceramides

ROC curve analysis was conducted to evaluate the discrimination of Mehran risk score and ceramides for AKI (Table 3). These ceramides achieved an AUC of 0.581–0.661 (P < 0.001) for AKI, and the AUC of the Mehran risk score was 0.780 (95% CI 0.738–0.821, P < 0.001). In order to evaluate the additional prognostic value of ceramides with respect to AKI risk beyond the Mehran risk score, models of each ceramide combined with the Mehran risk score were calculated and achieved an AUC of 0.802–0.808, which was significantly greater than that of the Mehran risk score alone (P < 0.05, Table 3). Moreover, the AUC of the ratio of the 10 ceramides to Cer(d18:1–24:0) for patients with ACS was 0.550–0.738, significantly larger than that of the 10 ceramides alone (Additional file 2: Table S3). Notably, the ratio of Cer(d18:1–24:2) to Cer(d18:1–24:0) had the highest AUCs among the ceramides.

**Table 3:** Predictive value of ceramides and Mehran risk score for acute kidney injury

Predictors	AUC	95%CI	*P* for AUC	*P* for AUC comparison
Mehran risk score	0.780	0.738–0.821	< 0.001	Reference
Cer(d18:1–16:0)	0.619	0.564–0.673	< 0.001	< 0.001
Cer(d18:1–18:0)	0.641	0.589–0.693	< 0.001	< 0.001
Cer(d18:1–20:0)	0.581	0.525–0.637	0.003	< 0.001
Cer(d18:1–21:0)	0.609	0.555–0.662	< 0.001	< 0.001
Cer(d18:1–24:1)	0.618	0.564–0.671	< 0.001	< 0.001
Cer(d18:1–24:2)	0.661	0.614–0.708	< 0.001	< 0.001
Cer(d18:1–16:0) plus MRS	0.807	0.768–0.845	< 0.001	0.021
Cer(d18:1–18:0) plus MRS	0.808	0.770–0.847	< 0.001	0.006
Cer(d18:1–20:0) plus MRS	0.805	0.766–0.843	< 0.001	0.038
Cer(d18:1–21:0) plus MRS	0.802	0.764–0.840	< 0.001	0.031
Cer(d18:1–24:1) plus MRS	0.805	0.766–0.844	< 0.001	0.025
Cer(d18:1–24:2) plus MRS	0.805	0.768–0.843	< 0.001	0.009

The areas under the curves (AUCs) were analyzed by receiver operating characteristic curves. The model of ceramides combined with Mehran risk score was calculated by logistic regression

*MRS* Mehran risk score, *CI* confidence interval

## Discussion

In this study, the incidence of AKI in patients with ACS was 16.5%, similar to those in previous meta-analysis reports (15.8%) and our previous retrospective study [41, 42]. We tested 11 ceramides using targeted LC–MS/MS; the chain lengths ranged from 16 to 26. Patients with AKI had higher levels of long-chain ceramides (C16:0, C18:0, C20:0, and C21:0) and very long-chain ceramides (C24:1 and C24:2). Furthermore, in risk-factor adjusted logistic models, these ceramides were independently associated with AKI events in patients with both STEMI and NSTE-ACS. Per Spearman’s correlation analysis, these ceramides positively correlated with the ACS risk score, contrast-induced nephropathy risk score, cardiac marker, baseline renal function, stress marker, and inflammatory markers, all of which may be potential contributing factors to AKI.

Previous studies have confirmed the relationship between ceramides, cardiovascular disease progression, and adverse events [7–10]. However, most studies only focused on Cer(d18:1/16:0), Cer(d18:1/18:0), Cer(d18:1/24:0), and Cer(d18:1/24:1) [5, 9, 43]. To our knowledge, we provided a wider spectrum of ceramides in cardiovascular research; we identified six ceramides related to AKI. Moreover, incremental predictive power was shown when ceramides were standardised to Cer(d18:1/24:0) [7–10, 44], which is highly abundant and stable in plasma and unaffected by the disease. Consistent with previous reports, ceramides standardised to Cer(d18:1/24:0) had a higher OR and AUC, especially Cer(d18:1/24:2).

Given that ACS patients are often exposed to contrast agents during treatment, the Mehran risk score for the prediction of contrast-induced nephropathy after PCI was established in 2004 [37]. Similar to previous studies, the AUC of the Mehran risk score was 0.78 in this study [45, 46]. Although ceramides alone demonstrated a poor discrimination power for AKI, when combined with the Mehran risk score, the AUC could significantly improve over 0.80. These results suggested that ceramides significantly increase the predictive effect of AKI beyond traditional scoring. It may be because they are involved in other mechanisms of AKI. According to a review by Ueda, ceramides are synthesised under stimulation and lead to apoptosis in renal tubular cells through mitogen-activated protein kinases signal [16]. Furthermore, a major precondition of AKI is producing reactive oxygen species [47]. In vivo and in vitro studies have explored the role of ceramide triggers in generating reactive oxygen species and the increase in oxidative stress that leads to endothelial dysfunction and renal function injury [14, 48]. More importantly, one study showed that plasma ceramide from other tissues and organs might also cause kidney damage [30]; upregulated ceramide levels in ACS patients may have derived from the cardiovascular system and led to AKI. Additionally, some studies found that patients with chronic kidney disease had elevated plasma ceramide levels [28, 29]. Previous studies found that long-chain (C16, C22) and very long-chain (C24) ceramides accounted for 20%, 10%, and 70%, respectively, of the amount of ceramides in the renal cortex [49]. In plasma, very long chain ceramides (C24) also have the highest mass fraction (approximately 30%), and Cer(d18:1/24:2) normalised to Cer(d18:1/24:0) had the best performance for AKI prediction. These results suggested that very long-chain ceramides (C24) may be key bioactive substances in the pathophysiology of AKI.

The application prospect of ceramides as biomarkers of AKI is that it may measure risk and guide interventions. Although randomised controlled trials regarding ceramides have not been reported, some studies have found that plasma ceramide levels could be reduced following aerobic exercise and statin therapy [12, 50]. While exercise and lipid-lowering treatment can also prevent the occurrence of AKI [51, 52], it means that the lowering of ceramide levels can be achieved through efficient lifestyle counselling and optimal administration of statin doses, ezetimibe combinations, and novel therapies such as PCSK9 inhibitors and may be an effective protective measure against AKI. Additionally, many clinical laboratories in modern medical institutions have automatic sample processing systems and mass spectrometry equipment. Therefore, in high-throughput QC environments, the measurement of plasma ceramides could be direct and cost-effective. Nevertheless, a sample run with the method used in the present study takes 5 min, allowing only about 20 samples to be run in an hour, making it unsuitable for large-scale clinical screening of AKI, but it could be kept for selected patients. In addition, it could be possible to optimize the technique. The present study provides data for the involvement of long-chain and very-long-chain ceramides in the development of AKI after ACS. Whether these ceramides are markers of AKI development or factors involved in AKI development remains to be investigated. More research is needed to explore the relationship between ceramides in cardiovascular disease and kidney disease, as well as the interaction between them. Additionally, there are many kinds of ceramides, and their biological activities in diseases are different, which may be related to different synthetic pathways. In a word, the implications of having a deep understanding of ceramide subtypes are significant.

### Limitations

First, although this was a multicentre, prospective study, owing to the relatively small sample size, additional studies are required to confirm these findings. Second, the first creatinine measure on admission was used as the baseline creatinine value, which may have been higher than the actual baseline creatinine for patients with ACS, even though only patients with onset time < 24 h were included; thus, the incidence of AKI may be underestimated, and the causality between ceramides and AKI remains inconclusive. Third, a control group of patients with CKD who did not develop AKI would have been useful, but the available data did not allow such analyses. Fourth, data for subgrouping AKI were not available. Fifth, although statins were administered to almost all patients with acute chest pain, this study did not collect data on the history of statin use, which may have impacted ceramide levels and the risk of AKI. Sixth, C12:0, C14:0, and > C26:0 ceramides were not examined in the present study, and whether they could be used for diagnosing AKI remains unknown. Seventh, ceramides have been associated with heart remodelling [11, 13], but no heart remodelling parameters were available. Finally, the role of metabolites, such as sphingosine, in ceramide synthetic pathways and carriers of ceramide, such as lipoprotein, and their relation to AKI were not studied.

## Conclusion

Long-chain ceramides (C16:0, C18:0, C20:0, and C21:0) and very long-chain ceramides (C24:1 and C24:2) were independently associated with the risk of AKI. These ceramides have a significant additional predictive value for AKI beyond traditional risk assessment tools. The ratio of Cer(d18:1/24:2) to Cer(d18:1/24:0) may be an indicator to identify AKI risk and guide effective clinical prediction, prevention, and intervention. The function of ceramide subtypes in cardiorenal diseases should be further researched.

## Disclosure

The authors report no disclosures relevant to the manuscript.

## Supplementary Information

**Additional file 1: Figure S1.** (A) Correlation analysis among all eleven ceramides. The value in the heat map is the correlation coefficient. ^*^ < 0.05; ^**^ < 0.001; ^***^ < 0.0001. (B) The average mass fraction of all eleven ceramides in ACS patients.

**Additional file 2: Table S1.** Relationships between ceramides and acute kidney injury in patients with STEMI and NSTE-ACS according to multivariate Logistic regression analysis. **Table S2.** Relationships between ratio of ten ceramides to Cer(d18:1–24:0) and acute kidney injury in patients with acute coronary syndrome. **Table S3.** Predictive value of ratio of ten ceramides to Cer(d18:1–24:0) for patients with acute coronary syndrome.

## Data Availability

The data underlying this article will be shared on reasonable request to the corresponding author.
