# Supplementary figures and images for: Long- and very long-chain ceramides are predictors of acute kidney injury in patients with acute coronary syndrome: the PEACP study
Source: Cardiovasc Diabetol. 2023 Apr 20;22:92. doi: 10.1186/s12933-023-01831-6 (PMC10120114; doi:10.1186/s12933-023-01831-6)

## Slide 1
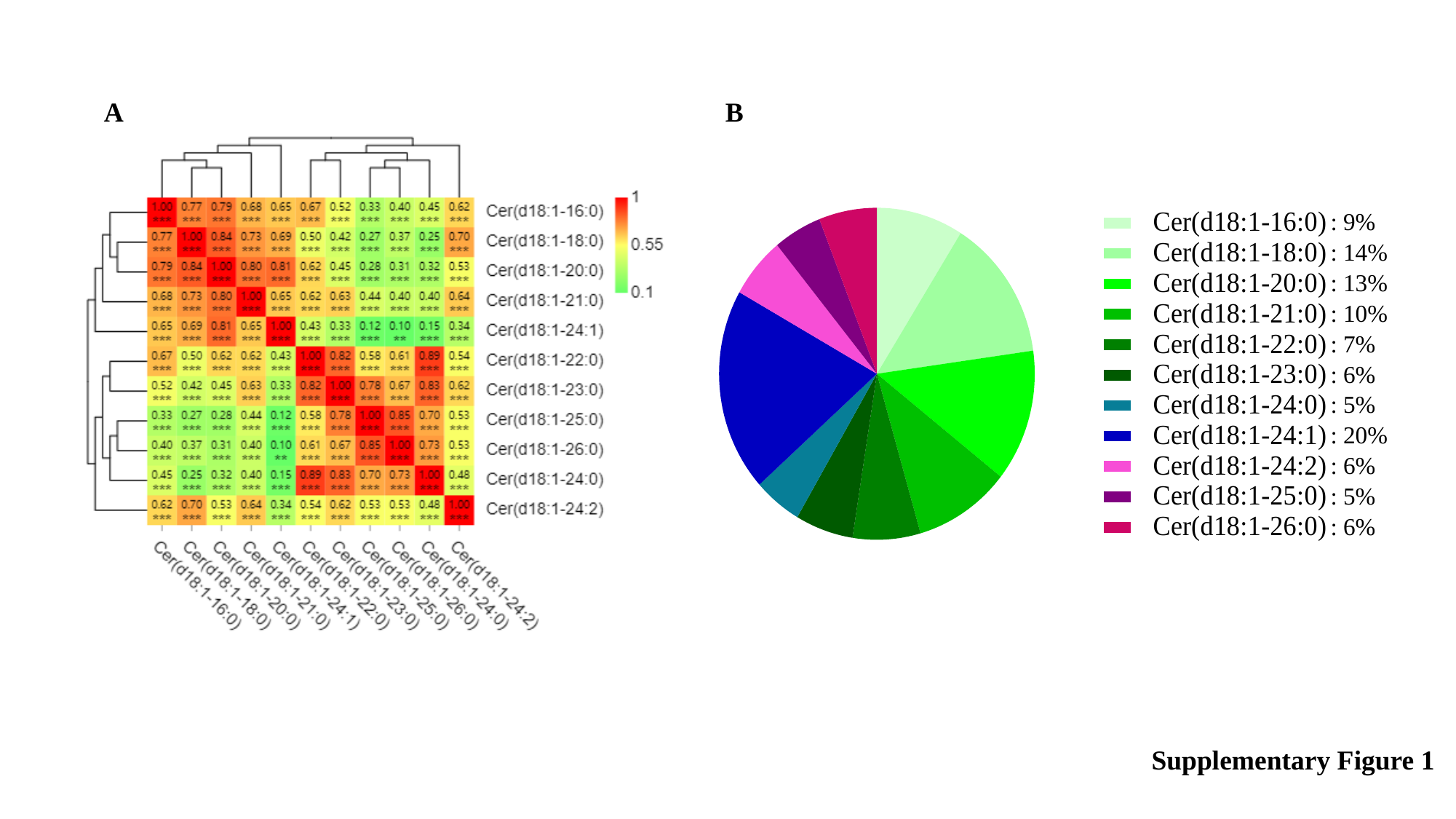

A
B
| : 9% |
| --- |
| : 14% |
| : 13% |
| : 10% |
| : 7% |
| : 6% |
| : 5% |
| : 20% |
| : 6% |
| : 5% |
| : 6% |
Supplementary Figure 1

Supplement: Supplementary file 1 — Additional file 1: Figure S1. (A) Correlation analysis among all eleven ceramides. The value in the heat map is the correlation coefficient. * < 0.05; ** < 0.001; *** < 0.0001. (B) The average mass fraction of all eleven ceramides in ACS patients. [file 12933_2023_1831_MOESM1_ESM.pptx]
