# Supplementary material for: Long- and very long-chain ceramides are predictors of acute kidney injury in patients with acute coronary syndrome: the PEACP study
Source: Cardiovasc Diabetol. 2023 Apr 20;22:92. doi: 10.1186/s12933-023-01831-6 (PMC10120114; doi:10.1186/s12933-023-01831-6)
Supplement: Supplementary file 2 — Additional file 2: Table S1. Relationships between ceramides and acute kidney injury in patients with STEMI and NSTE-ACS according to multivariate Logistic regression analysis. Table S2. Relationships between ratio of ten ceramides to Cer(d18:1–24:0) and acute kidney injury in patients with acute coronary syndrome. Table S3. Predictive value of ratio of ten ceramides to Cer(d18:1–24:0) for patients with acute coronary syndrome. [file 12933_2023_1831_MOESM2_ESM.docx]

Additional file Table 1. Relationships between ceramides and acute kidney injury in patients with STEMI and NSTE-ACS according to multivariate Logistic regression analysis.

| **Ceramides** | **OR (95%CI)**  **per SD** | **P** |  | **OR (95%CI)**  **Q4 vs. Q1** | **P** |
| --- | --- | --- | --- | --- | --- |
| **NSTE-ACS** |  |  |  |  |  |
| Cer(d18:1-16:0) | 1.559 (1.144-2.124) | **0.005** |  | 2.173 (1.185-5.340) | **0.041** |
| Cer(d18:1-18:0) | 1.384 (1.064-1.799) | **0.015** |  | 2.715 (1.158-6.365) | **0.022** |
| Cer(d18:1-20:0) | 1.408 (1.057-1.875) | **0.019** |  | 1.030 (0.452-2.345) | 0.944 |
| Cer(d18:1-21:0) | 1.412 (1.034-1.927) | **0.030** |  | 2.321 (1.055-5.641) | **0.048** |
| Cer(d18:1-22:0) | 1.064 (0.753-1.503) | 0.725 |  | 1.133 (0.473-2.716) | 0.780 |
| Cer(d18:1-23:0) | 1.272 (0.925-1.751) | 0.139 |  | 1.836 (0.781-4.320) | 0.164 |
| Cer(d18:1-24:0) | 0.906 (0.634-1.295) | 0.590 |  | 0.902 (0.375-2.170) | 0.818 |
| Cer(d18:1-24:1) | 1.510 (1.121-2.034) | **0.007** |  | 2.076 (0.936-4.606) | 0.072 |
| Cer(d18:1-24:2) | 1.510 (1.169-1.950) | **0.002** |  | 3.606 (1.596-8.147) | **0.002** |
| Cer(d18:1-25:0) | 1.196 (0.891-1.606) | 0.233 |  | 1.604 (0.703-3.663) | 0.262 |
| Cer(d18:1-26:0) | 1.145 (0.890-1.472) | 0.294 |  | 0.826 (0.357-1.907) | 0.654 |
| **STEMI** |  |  |  |  |  |
| Cer(d18:1-16:0) | 1.596 (1.033-2.467) | **0.035** |  | 2.173 (0.885-5.340) | 0.091 |
| Cer(d18:1-18:0) | 1.673 (1.096-2.555) | **0.017** |  | 4.118 (1.148-14.769) | **0.030** |
| Cer(d18:1-20:0) | 1.583 (1.048-2.393) | **0.029** |  | 1.665 (0.557-4.973) | 0.361 |
| Cer(d18:1-21:0) | 1.729 (1.175-2.544) | **0.005** |  | 2.737 (1.095-7.880) | **0.046** |
| Cer(d18:1-22:0) | 1.082 (0.640-1.827) | 0.769 |  | 0.645 (0.167-2.496) | 0.525 |
| Cer(d18:1-23:0) | 1.077 (0.689-1.685) | 0.743 |  | 0.845 (0.261-2.741) | 0.779 |
| Cer(d18:1-24:0) | 0.909 (0.537-1.540) | 0.724 |  | 0.640 (0.189-2.164) | 0.473 |
| Cer(d18:1-24:1) | 1.355 (0.884-2.078) | 0.164 |  | 1.978 (0.647-6.051) | 0.232 |
| Cer(d18:1-24:2) | 1.759 (1.206-2.565) | **0.003** |  | 6.464 (1.553-26.903) | **0.010** |
| Cer(d18:1-25:0) | 1.254 (0.833-1.888) | 0.279 |  | 1.097 (0.380-3.168) | 0.864 |
| Cer(d18:1-26:0) | 1.285 (0.857-1.927) | 0.225 |  | 1.242 (0.442-3.486) | 0.681 |

AKI, acute kidney injury; STEMI, ST-elevation elevated myocardial infarction; NSTE-ACS, non-ST-elevation acute coronary syndrome; OR, odds ratio; CI, confidence interval. Models were adjusted by age, sex (male vs. female), systolic blood pressure, heart rate, body mass index, statin (yes vs. no), hypertension (yes vs. no), diabetes (yes vs. no), hyperlipidemia (yes vs. no), Killip classification, white blood cell count, low density lipoprotein cholesterol, total cholesterol, triglyceride, estimated glomerular filtration rate, high sensitive cardiac troponin T, N-terminal pro-brain natriuretic peptide, and Mehran risk score.

Additional file Table 2. Relationships between ratio of ten ceramides to Cer(d18:1-24:0) and acute kidney injury in patients with acute coronary syndrome.

| **Ceramides** | **OR (95%CI)**  **per SD** | **P** |  | **OR (95%CI)**  **Q4 vs. Q1** | **P** |
| --- | --- | --- | --- | --- | --- |
| Cer(d18:1-16:0)/Cer(d18:1-24:0) | 1.450 (1.183-1.777) | **<0.001** |  | 2.118 (1.096-4.094) | **0.026** |
| Cer(d18:1-18:0)/Cer(d18:1-24:0) | 1.499 (1.222-1.840) | **<0.001** |  | 2.971 (1.539-5.737) | **0.001** |
| Cer(d18:1-20:0)/Cer(d18:1-24:0) | 1.383 (1.137-1.682) | **0.001** |  | 2.263 (1.171-4.373) | **0.015** |
| Cer(d18:1-21:0)/Cer(d18:1-24:0) | 1.435 (1.167-1.765) | **0.001** |  | 2.659 (1.364-5.184) | **0.004** |
| Cer(d18:1-22:0)/Cer(d18:1-24:0) | 1.267 (1.034-1.553) | **0.022** |  | 1.293 (0.698-2.396) | 0.414 |
| Cer(d18:1-23:0)/Cer(d18:1-24:0) | 1.442 (1.176-1.768) | **<0.001** |  | 2.866 (1.46-5.626) | **0.002** |
| Cer(d18:1-24:1)/Cer(d18:1-24:0) | 1.331 (1.101-1.608) | **0.003** |  | 1.754 (0.854-3.601) | 0.126 |
| Cer(d18:1-24:2)/Cer(d18:1-24:0) | 1.755 (1.429-2.155) | **<0.001** |  | 7.678 (3.513-16.781) | **<0.001** |
| Cer(d18:1-25:0)/Cer(d18:1-24:0) | 1.343 (1.091-1.653) | **0.005** |  | 3.239 (1.671-6.278) | **0.001** |
| Cer(d18:1-26:0)/Cer(d18:1-24:0) | 1.260 (1.036-1.533) | **0.021** |  | 1.904 (1.044-3.471) | **0.036** |

AKI, acute kidney injury; OR, odds ratio; CI, confidence interval. Models were adjusted the same as Additional file Table 1

Additional file Table 3. Predictive value of ratio of ten ceramides to Cer(d18:1-24:0) for patients with acute coronary syndrome.

| **Predictors** | **AUC** | **95%CI** | **P for AUC** | **Reference** | **P for AUC comparison** |
| --- | --- | --- | --- | --- | --- |
| Cer(d18:1-16:0)/Cer(d18:1-24:0) | 0.674 | 0.625-0.723 | <0.001 | Cer(d18:1-16:0) | <0.001 |
| Cer(d18:1-18:0)/Cer(d18:1-24:0) | 0.684 | 0.635-0.734 | <0.001 | Cer(d18:1-18:0) | <0.001 |
| Cer(d18:1-20:0)/Cer(d18:1-24:0) | 0.640 | 0.589-0.69 | <0.001 | Cer(d18:1-20:0) | <0.001 |
| Cer(d18:1-21:0)/Cer(d18:1-24:0) | 0.670 | 0.622-0.718 | <0.001 | Cer(d18:1-21:0) | <0.001 |
| Cer(d18:1-22:0)/Cer(d18:1-24:0) | 0.590 | 0.537-0.642 | 0.001 | Cer(d18:1-22:0) | <0.001 |
| Cer(d18:1-23:0)/Cer(d18:1-24:0) | 0.660 | 0.612-0.709 | <0.001 | Cer(d18:1-23:0) | <0.001 |
| Cer(d18:1-24:1)/Cer(d18:1-24:0) | 0.645 | 0.596-0.695 | <0.001 | Cer(d18:1-24:1) | <0.001 |
| Cer(d18:1-24:2)/Cer(d18:1-24:0) | 0.738 | 0.694-0.782 | <0.001 | Cer(d18:1-24:2) | <0.001 |
| Cer(d18:1-25:0)/Cer(d18:1-24:0) | 0.596 | 0.544-0.649 | <0.001 | Cer(d18:1-25:0) | <0.001 |
| Cer(d18:1-26:0)/Cer(d18:1-24:0) | 0.550 | 0.496-0.604 | 0.062 | Cer(d18:1-26:0) | 0.001 |

The area under the curves (AUC) were analyzed by receiver operating characteristic curves. CI, confidence interval.
